# Structures of monomeric and oligomeric forms of the *Toxoplasma gondii* perforin-like protein 1

**DOI:** 10.1126/sciadv.aaq0762

**Published:** 2018-03-21

**Authors:** Tao Ni, Sophie I. Williams, Saša Rezelj, Gregor Anderluh, Karl Harlos, Phillip J. Stansfeld, Robert J. C. Gilbert

**Affiliations:** 1 Division of Structural Biology, Wellcome Trust Centre for Human Genetics, University of Oxford, Roosevelt Drive, Oxford OX3 7BN, UK.; 2 Department of Biochemistry, University of Oxford, South Parks Road, Oxford OX1 3QU, UK.; 3 Department of Molecular Biology and Nanobiotechnology, National Institute of Chemistry, Hajdrihova 19, 1000 Ljubljana, Slovenia.

## Abstract

*Toxoplasma* and *Plasmodium* are the parasitic agents of toxoplasmosis and malaria, respectively, and use perforin-like proteins (PLPs) to invade host organisms and complete their life cycles. The *Toxoplasma gondii* PLP1 (*Tg*PLP1) is required for efficient exit from parasitophorous vacuoles in which proliferation occurs. We report structures of the membrane attack complex/perforin (MACPF) and Apicomplexan PLP C-terminal β-pleated sheet (APCβ) domains of *Tg*PLP1. The MACPF domain forms hexameric assemblies, with ring and helix geometries, and the APCβ domain has a novel β-prism fold joined to the MACPF domain by a short linker. Molecular dynamics simulations suggest that the helical MACPF oligomer preserves a biologically important interface, whereas the APCβ domain binds preferentially through a hydrophobic loop to membrane phosphatidylethanolamine, enhanced by the additional presence of inositol phosphate lipids. This mode of membrane binding is supported by site-directed mutagenesis data from a liposome-based assay. Together, these structural and biophysical findings provide insights into the molecular mechanism of membrane targeting by *Tg*PLP1.

## Teaser

Structures of *Toxoplasma* perforin-like protein facilitate understanding of the protein’s role in infection and disease.

## INTRODUCTION

Up to one-third of the global population is thought to be infected with *Toxoplasma gondii*, a zoonotic agent of disease that can be fatal in developing fetuses and the immunocompromised (*1*, *2*). Infection commonly manifests in fever, headache, myalgia, and anorexia (*3*). Chronic infection by *Toxoplasma* is believed to underlie forms of psychiatric pathology (*4*, *5*). *T. gondii* has a heteroxenous life cycle in which an obligate sexual feline stage of parasitism is coupled to asexual reproduction in a nonfeline host (*2*, *3*, *5*, *6*). The cell forms associated with asexual proliferation of parasites include primarily the tachyzoite, but others are cyst-forming bradyzoites and merozoites that expand the parasite load in the cat intestine. Tachyzoites invade and reproduce within host cells by the formation of a parasitophorous vacuole (PV) from host cell membranes within which they can grow in a protected environment. Escape from the PV, and therefore from the infected cell, for subsequent rounds of infection has been shown to be dependent on the perforin-like protein *Tg*PLP1 (*T. gondii* PLP) (*7*–*9*).

So far, little information has been available concerning the structure and mechanism of *Tg*PLP1. Within *T. gondii* genome sequences, the ratio of nonsynonymous/synonymous single-nucleotide polymorphisms (SNPs) for *Tg*PLP1 is 0.1 (24 of 233 SNPs, 64 genomes), indicating that it is one of the better-conserved *Toxoplasma* proteins (http://toxodb.org/toxo/). Sequence analysis reveals that apicomplexan perforin-like proteins (ApiPLPs) generally have a conserved core membrane attack complex/perforin (MACPF) domain followed by a C-terminal β-strand–rich domain [CTD; dubbed Apicomplexan PLP C-terminal β-pleated sheet (APCβ)] and complemented by N-terminal domains (NTDs) (fig. S1), which vary greatly in length and may have significant regions of intrinsic disorder (*10*).

The MACPF domains of the apicomplexan PLPs are typical of members of the perforin branch of the MACPF/cholesterol-dependent cytolysin (MACPF/CDC) superfamily of pore-forming and membrane-targeting proteins (*10*, *11*). The core feature of the characteristic MACPF/CDC domain is a bent four-stranded antiparallel β sheet with a set of α helices suspended between each pair of strands. In all pore-forming MACPF/CDC proteins characterized to date, oligomerization leads to conversion of these α helices to form a β sheet [made of transmembrane hairpins (TMHs)] that continues the domain core (*12*–*14*). The recently solved structure of human astrotactin-2, a protein involved in cell migration and polarity, revealed a perforin-like protein with mismatched TMH regions, suggesting that it is unlikely to be involved in pore formation in the way that homologous proteins are (fig. S1C) (*11*, *15*). By contrast, sequence analysis reveals that *Tg*PLP1 and the other apicomplexan PLPs have TMH regions that would be expected to play a role in membrane insertion during pore formation or another type of membrane-targeting mechanism.

Compared with known features of *Tg*PLP1 MACPF domains, the structure and function of the APCβ domain of *Tg*PLP1 are less well characterized. It is representative of a homologous domain possessed by all ApiPLPs but has an unknown structure (*10*). Similar to the CTDs of perforin itself (a C2 domain) and of the CDCs [immunoglobulin (Ig)–like domains], which are structurally homologous to each other (*16*), it is predicted to be β-sheeted, although built from three copies of a common motif. Both the NTDs and CTDs (APCβ domain) of *Tg*PLP1 have been reported to have a role in membrane binding before pore formation, with the APCβ domain playing the primary and essential role (*8*). Here, we report the structures of the MACPF domain and APCβ domain of *Tg*PLP1, alone and in tandem, and of two different MACPF domain oligomers. Combined with functional data, molecular dynamics (MD) simulations, and mutational analysis, these structural studies provide insights into the molecular mechanism of *Tg*PLP1 membrane activity.

## RESULTS

### Crystal structures of *Tg*PLP1 MACPF domain

We found that the transient expression of *Tg*PLP1 in mammalian cells (*17*) yields ample protein for structural and biophysical studies (see Materials and Methods). We first obtained oligomeric crystal forms of the *Tg*PLP1 MACPF domain by the use of in situ trypsinization in crystallization drops. On solution of the resulting structures, we found two related assemblies: a ring-shaped hexamer and a helical packing arrangement with six subunits per turn. The helical subunit arrangement was formed in a crystal of space group *P*6_5_ that diffracted to 2.03 Å with phasing using anomalous scattering from a single platinum derivative (see Materials and Methods). The ring-shaped assembly was, by contrast, found in a *C*222_1_ crystal form that diffracted to only 5.0 Å and that we phased by molecular replacement using a single subunit from the *P*6_5_ crystal form. Molecular replacement in the same way was also used to solve the structure of an intact monomeric form of the MACPF domain at 3.11 Å in space group *C*2.

**Fig. 1:**
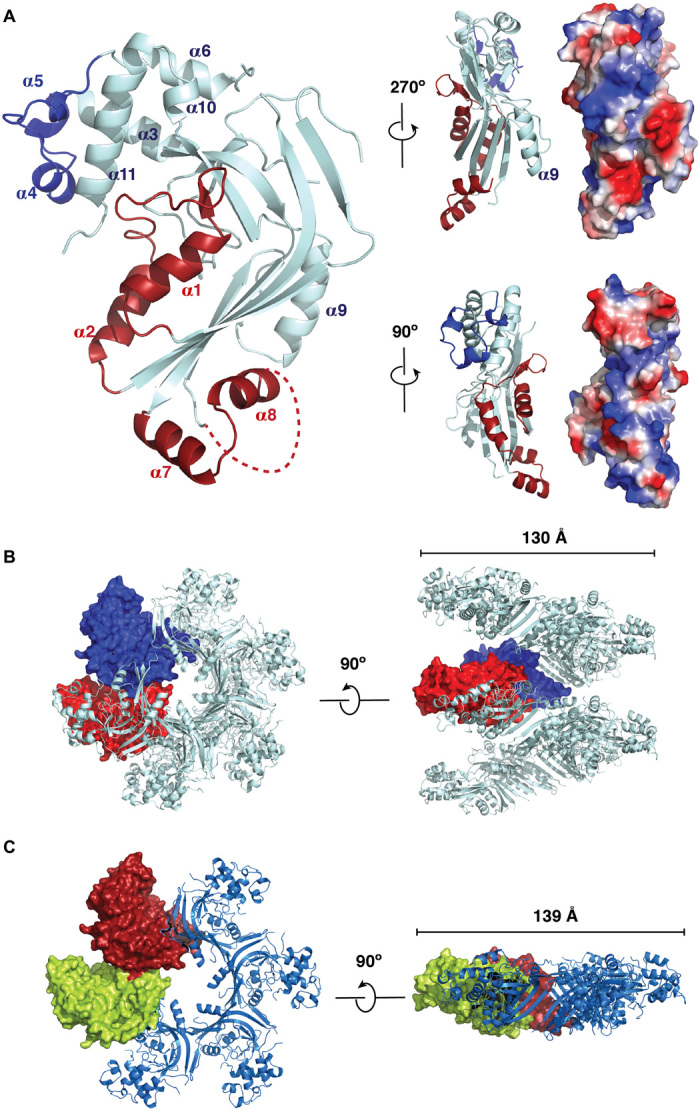
Crystal structure of *Tg*PLP1 MACPF domain. (**A**) Overall structure of *Tg*PLP1 MACPF domain. Left: The α helices are labeled from α1 to α9 from N to C terminus of the MACPF domain, the TMHs (α1 to α2 and α7 to α8) are highlighted in red, and the additional α helices (α4 to α5) compared with perforin-1 MACPF domain are colored in blue. Right: Side views of *Tg*PLP1 MACPF domain showing the angled α9 and the surface charge distribution. (**B**) Helical assembly of the *Tg*PLP1 MACPF domain. Top and side views of helical assembly of MACPF domain are shown, with two protein surfaces presented in red and blue. (**C**) Ring assembly of *Tg*PLP1 MACPF domain. Top and side views of ring assembly of MACPF domain are shown, with two protein surfaces presented in red and green. The relative positions of proteins in the helical and ring assemblies are compared, and the diameter of the helix and ring are shown.

For clarity, we describe features of the isolated intact MACPF domain first. In addition to the core-bent β sheet, we were able to resolve the whole of the TMH1 region (helices α1 and α2) and two parts of the TMH2 region (helices α7 and α8) (Fig. 1A), although the TMH2 region was partly disordered and thus not visible in the crystal structure. Whereas TMH1 is packed into a pocket, TMH2 is more exposed to solvent, explaining its mobility. In addition to a further set of helices that cap the top of the core β sheet, we noted two helical inserts (α4 and α5) not present in other MACPF domains to have had their structures resolved and that α9 is angled out from the core of the domain (Fig. 1A). Together, these features cause the *Tg*PLP1 MACPF domain to be somewhat bulkier than other perforin-like proteins, which partly explains its capacity to form much smaller oligomers than other family members (see below).

The helical oligomeric form displays excellent geometry in which the central β-strands from adjacent subunits form a continuous β sheet with six hydrogen bonds between each strand (Fig. 1B and fig. S2A). Critically, the helical assembly can only form crystallographically with the trypsinization of TMH2, strongly suggesting that this is an oligomeric form related to that generated following membrane attack by *Tg*PLP1, because cutting away the helical TMH2 will mimic its transition to a β-stranded form (*13*, *14*, *18*). Biologically, no cleavage would occur, but the TMH2 region would move by spontaneous conversion to a membrane-inserted set of β-strands as part of *Tg*PLP1 activity.

The orientation of the subunits, with the TMH regions facing inward, is exactly what would be expected for the biological, and usually pore-forming, oligomer formed by MACPF/CDC proteins (*13*, *14*, *18*). In addition to the presence of α4 and α5 and the angle of α9, the subunits of *Tg*PLP1 are tilted sideways to allow closure around a narrow lumen with six subunits per turn (the smallest previously described MACPF/CDC oligomer is a dodecamer) (fig. S3) (*12*). Analysis with the software HOLE (*19*) indicates a minimum functional pore radius for the oligomeric state captured in the helix of 12.45 Å (fig. S3). We discuss the functional implications of this structure below, but this is the first crystal structure of a MACPF/CDC homo-oligomer in what is likely to reflect a membrane-active, and possibly pore-forming, state. By contrast, however, the ring-form oligomer crystal structure is imperfect with three main-chain hydrogen bonds being found between only two of the subunit interfaces (“A-B” and “D-E”). Together with the low resolution of the structure, which was only subject to rigid-body refinement, this suggests that the ring-form crystal structure may not represent a functional biological assembly although it may represent an assembly intermediate. The minimum functional pore radius for the hexameric ring, estimated as above, is 7.81 Å (Fig. 1C and fig. S3).

**Fig. 2:**
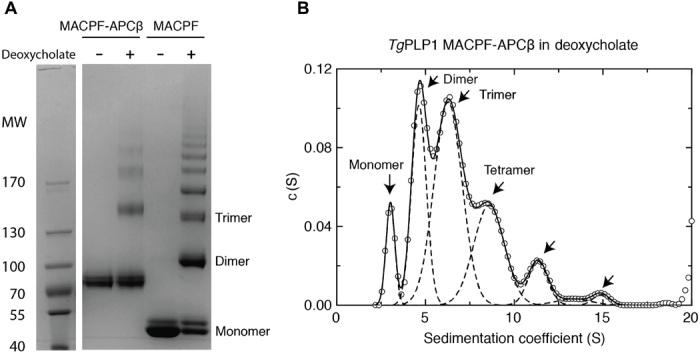
*Tg*PLP1 forms a detergent-induced oligomer. (**A**) SDS–polyacrylamide gel electrophoresis (SDS-PAGE) of *Tg*PLP1 MACPF-APCβ and MACPF domain with or without detergent. (**B**) Analytical ultracentrifugation of *Tg*PLP1 MACPF-APCβ (0.5 mg/ml) in detergent. The species corresponding to monomer, dimer, and other oligomers are indicated with arrows.

A number of pore-forming proteins have been shown to form their functional states in solution when concentrated or in the presence of detergent (*20*, *21*). For example, CDC pneumolysin forms helical oligomers in solution that preserve the subunit interface and conformation found in the planar ring–shaped pore (*14*, *18*). In line with this, the treatment of intact *Tg*PLP1 MACPF domains with the detergent deoxycholate results in their oligomerization (Fig. 2 and fig. S2B), and these oligomerized assemblies are capable of mediating transmembrane conductance (fig. S2C) when formed from full-length *Tg*PLP1. Conductance measurements of preformed *Tg*PLP1 oligomers in membranes show the formation of pores of variable size and stability and, eventually, membrane breakage (fig. S2C); these effects could be driven by a helical packing of *Tg*PLP1 subunits, which would be more likely to tear the membrane structure than a planar ring assembly (fig. S2C). However, we do not know the exact oligomeric form giving rise to pore formation and membrane breakage.

### MD simulations of MACPF domain oligomers

To investigate the functional significance of the MACPF domain oligomers further, we performed MD simulations of both the helical and ring-form crystal structures (fig. S4). Of most interest was the resilience of the different subunit interfaces observed. In the crystal structures, all subunit interfaces in the helical assembly are identical, but the ring oligomer has three different interfaces, with three, one, and no main-chain hydrogen bonds, respectively, in contrast to the six in each of the helical subunit interfaces. The hydrogen bonds observed in the helical assembly are in a different register to those found in the ring assembly. Over the course of a 100-ns simulation, the helical interfaces remained intact, whereas the interfaces for the ring underwent significant variation (figs. S5 and S6). To investigate whether the interfaces displayed more freedom when isolated from the oligomer as a whole, we then repeated the simulations but for dimers extracted from the complete assemblies (fig. S7), producing similar results to those observed for the simulations of the whole hexamer. Again, the ring interfaces underwent considerable changes, whereas the helical interface was maintained. The subunit interface captured in the helical oligomer could occur in a membrane-bound ring of six subunits, although in solution, it has relaxed into a helical arrangement. The ring assembly we observe in the low-resolution crystal form could be an intermediate on the way to the formation of such a helix, but its poor geometry and instability make it a poor candidate for a biologically active structure. It is also possible that *Tg*PLP1 assemblies of less than six subunits could be active on membranes; the functional significance of incomplete pore-forming rings of MACPF/CDC protein subunits is well attested (*22*–*25*). In all, these analyses suggest that the interfaces found in the helical assembly are more stable and of biological significance, although the resulting functional oligomer remains to be determined.

### Crystal structure of *Tg*PLP1 APCβ domain

**Fig. 3:**
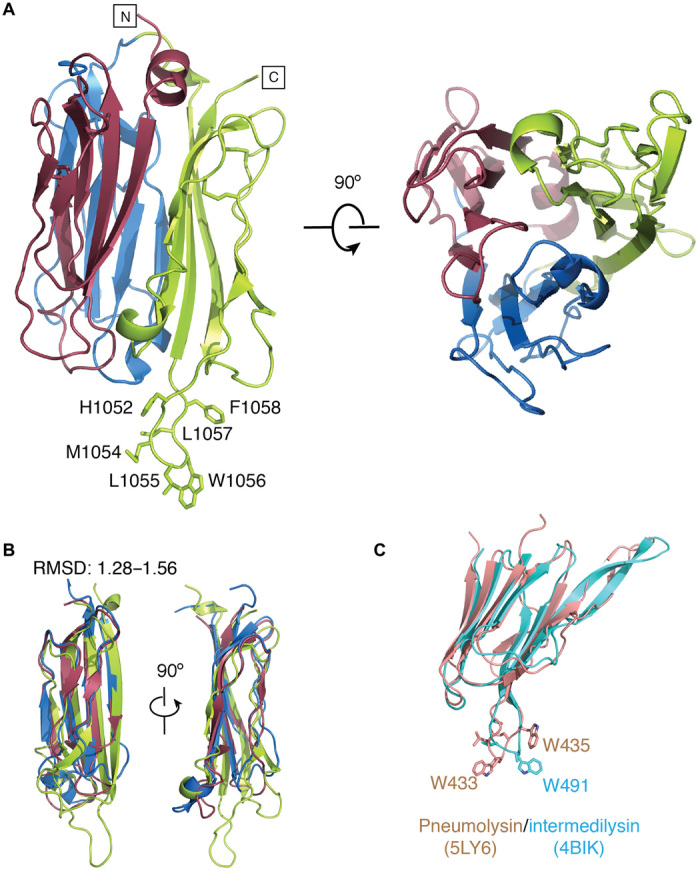
Crystal structure of *Tg*PLP1 APCβ domain. (**A**) Overall structure of APCβ domain with each repeat colored differently. Left: Side view of APCβ domain highlighting the hydrophobic tip at the bottom. Right: Top view showing threefold pseudosymmetry with four antiparallel β-strands forming the core of the domain. (**B**) Superposition of three tandem repeats showing the conservation within the main scaffold and variation in the bottom loop of each repeat. (**C**) Crystal structure of pneumolysin and intermedilysin Ig-like domains, with the tryptophan hydrophobic tip highlighted.

An expression construct representing the *Tg*PLP1 APCβ (residues 810 to 1072) was crystallized in space group *C*2 with the crystals diffracting to 1.1 Å resolution. Its structure (Fig. 3A and fig. S8, A and B) revealed the arrangement of three homologous repeats in a β-prism around a tightly packed and extremely hydrophobic (and aromatic) core. Comparison of the repeats with one another indicates a root mean square deviation (RMSD) of 1.28 to 1.56 Å over their roughly 80 residues each arranged into six β-strands with four forming one face of a sandwich and two the other (Fig. 3B). The two sides of the 4 + 2 sandwich forming each repeat are linked together by two highly conserved disulfide bonds, which can be treated as a signature motif of this domain (*26*). Comparison of the sequences of all available apicomplexan PLPs shows that sequences in the inner plane (four β-strands) facing toward the core are more conserved than in the outer set (two β-strands) (fig. S8C). Among the other notable features of the *Tg*PLP1 APCβ is the presence of a long projecting loop at its base, which is tipped by a tryptophan and has additional sequence features that make it resemble the similar loops found at the base of the CTDs of perforin itself and the CDCs (Fig. 3C) (*16*). However, the topology of each repeat in the β-prism is different from those of the perforin and CDC CTDs (which are similar to one another), and therefore, this common feature in *Tg*PLP1 appears to be a product of convergent and not divergent evolution (fig. S8D).

### MD simulations of *Tg*PLP1 APCβ domain interacting with membrane

**Fig. 4:**
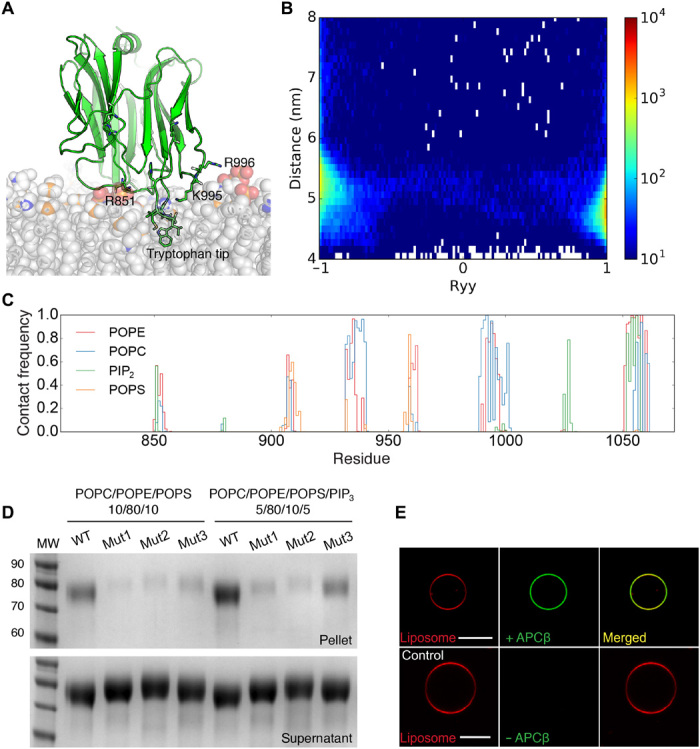
Characterization of *Tg*PLP1 APCβ membrane binding using MD simulations and liposome sedimentation assays. (**A**) A snapshot from a 100-ns all-atom (AT) MD simulation of *Tg*PLP1 APCβ domain binding to a lipid bilayer containing 45% POPC, 42% POPE, 10% POPS, 3% PIP_2_ showing the upright membrane-binding orientation with the extended loop inserted into the bilayer. Lipids are shown with carbon and oxygen colored in white, nitrogen in blue, phosphate in orange, and PIP_2_ head groups in red. (**B**) Density map showing APCβ membrane binding to a POPC/POPE/POPS/PIP_3_ membrane calculated from 20 coarse-grained simulations of 1 μs in length. The YY component of the rotational matrix of APCβ is shown versus the center of mass distance between APCβ and the lipid bilayer, indicating two inverse binding orientations of the domain. (**C**) Normalized average number of contacts between residues of the APCβ residues and each lipid type in the simulation, taken from three 100-ns AT simulations of APCβ with the POPC/POPE/POPS/PIP_2_ membrane. (**D**) Liposome sedimentation assay showing that the *Tg*PLP1 APCβ domain binds to membranes and mutations in the hydrophobic tryptophan tip and charge neutralization abolish membrane binding. Mutant 1 (mut1) affects the tryptophan loop tip (^1054^MLWL^1057^-AAAA); mut2 gives charge neutralization (R851A K995A R996A R1030A K1048A); mut3 affects an exposed histidine (H1052A). (**E**) Fluorescent images of GUVs labeled with rhodamine-phosphatidylethanolamine (red) interacting with His_6_-tagged APCβ labeled with green fluorescent anti-His_6_ antibody. Scale bar, 10 μm. WT, wild type.

To explore in detail the approach made by APCβ domains to membranes, we performed both coarse-grained and atomistic MD simulations (Fig. 4 and fig. S9). On the basis of comparison to other MACPF/CDC proteins with CTDs, we hypothesized that this would involve the surface of the domain containing the long loop tipped with a tryptophan approaching the membrane first. We found that in membranes composed of palmitoyloleoylphosphatidylethanolamine (POPE), palmitoyloleoylphosphatidylcholine (POPC), and palmitoyloleoylphosphatidylserine (POPS) lipids alone in various combinations, this was the case; however, also in the presence of phosphatidylinositol 4,5-bisphosphate (PIP_2_) or phosphatidylinositol 3,4,5-trisphosphate (PIP_3_), the domain bound upside-down about half the time (fig. S9, A and B) and associated with the membrane further from its surface. In the full-length protein, this upside-down binding mode is very unlikely because of the presence of the MACPF domain, which is only 11 residues before the first residue of the APCβ structure. This artifactual orientation in the MD simulation is an effect of the extra charge density associated with the presence of PIP lipids. Atomistic MD simulations were in agreement with the coarse-grained results and showed a definite preference for POPE enhanced by PIP_2_ (Fig. 4C and fig. S9C), whereas the tryptophan-tipped loop inserted into the hydrophobic core of the bilayer that helps to anchor the domain in the membrane.

To determine whether the orientation with the long loop down toward the membrane gives the correct interface for membrane binding, we used site-directed mutagenesis combined with liposome sedimentation assays (Fig. 4D). Complementary to our findings from MD simulations, we found that the wild-type protein had a preference for POPE and also for PIP_3_-containing membranes, but that binding is reduced by mutation of the extended tip (MLWL→AAAA), by mutation of positively charged residues to neutral ones on the bottom of the domain (K→A, R→A), and by mutation of a histidine in the loop to alanine. The MLWL→AAAA and Lys/Arg→Ala mutants had a greater effect on binding than the His→Ala mutation did, which indicates the primacy of charge-based and hydrophobic interactions for stable membrane association. Finally, we imaged the APCβ domain binding to the surface of POPE-containing giant unilamellar vesicles (GUVs) (Fig. 4E).

The structure of the MACPF-APCβ tandem domains of *Tg*PLP1 together was studied using small-angle x-ray scattering (SAXS) with modeling of the resulting data assisted by the shortness of the linker joining the MACPF and APCβ domains (fig. S10). The data indicate that the likely solution state of the MACPF-APCβ tandem is clustered in two conformations, in neither of which is there formed a close interface between the two domains.

## DISCUSSION

We have provided the first crystal structures for an ApiPLP, *Tg*PLP1. Crystal structures of the MACPF domain in oligomeric and monomeric states reveal the formation of a helical hexameric assembly that has a continuous intersubunit β sheet, and MD simulations and biophysical characterization suggest that the stable interface within the helical assembly is more likely to be of biological significance. The crystal structure of the C-terminal APCβ domain reveals a novel β-prism fold for the signature domain of the ApiPLPs. Together with MD calculations, site-directed mutagenesis, and fluorescent imaging, our data indicate that the membrane binding of APCβ depends on a projecting hydrophobic loop and positively charged residues surrounding it.

Our structural insights are of value for an understanding of the mechanisms used by ApiPLPs to facilitate parasite invasion and human disease and set the scene for further studies in which their role in the life cycles of their respective producing organisms will come to be further defined. The work presented here demonstrates that *Tg*PLP1 functions in a way different from any other perforin-like protein so far described. The existing data on *Tg*PLP1 oligomerization leave open questions concerning the details of its mechanism of action. For now, different possibilities exist, which include subunits assembling in a helical arrangement to disrupt membranes directly, rings or arcs of subunits forming on membranes, or another kind of functional assembly. Although our data strongly support a biological role for the interface captured in the helical oligomer, this will require further verification. Our data show that *Tg*PLP1 does not form large ring-like oligomers on membranes like other MACPF/CDC pore-forming proteins, but rather smaller assemblies. It suggests that the role of *Tg*PLP1 is not to directly generate a large and stable opening through the PV membrane, such as those formed by MACPF/CDC proteins like perforin-1 or the CDCs, but is either to weaken the membrane for mechanical disruption by parasites or to allow the delivery of small effector molecules from the PV itself into the cytoplasm or vice versa. The active state could be a complete helical turn or ring of subunits, or perhaps more likely a smaller, arc-shaped assembly (*23*–*25*). Arcs of *Tg*PLP1 subunits could function in isolation or together, in which case they could create patchwork assemblies such as those formed by the larger arcs of CDC listeriolysin subunits (*25*, *27*). In any case, our work establishes the molecular basis for an understanding of the mechanism of action of a class of MACPF/CDC proteins critical in some of the world’s most pernicious and debilitating diseases caused by apicomplexan parasites.

## MATERIALS AND METHODS

### Construct cloning and site-directed mutagenesis

*Tg*PLP1 MACPF domain (residues 462 to 809, N720Q/N744Q) was cloned into the mammalian protein expression vector pHLsec by inserting the DNA fragment between Age I and Kpn I restriction sites, resulting in a signal peptide in the N terminus of the proteins and a KTHHHHH tag at the C terminus of the protein. The signal peptide was cleaved during protein secretion into the media, leaving an additional three residues Glu-Thr-Gly at the N terminus of the proteins. The APCβ domain (residues 810 to 1072) and the MACPF-APCβ domain tandem (residues 462 to 1072) constructs were cloned in the same way. For site-directed mutagenesis, overlapping polymerase chain reaction was performed.

### Protein expression, purification, and crystallization

All the *Tg*PLP1 proteins used in this study were expressed in mammalian human embryonic kidney (HEK) 293T cells according to expression protocols previously reported (*17*). The protein expression screen was initially carried out in *Escherichia coli* with its respective expression vectors (pOPINE, pOPINF, pOPINM, and pOPINJ), resulting in an insoluble aggregate preventing further biophysical characterization, even after the extensive optimization of expression tags and purification conditions.

To express the *Tg*PLP1 constructs in HEK293T cells, 6 mg of purified DNA was transfected into 12 roller bottles of HEK293T cells in the presence of the glycosylation inhibitor kifunensine (final concentration of 5 μM) with 12 mg of polyethylenimine. The transfected cells were harvested 4 to 5 days after transfection. Cell media (3 liters) containing the secreted proteins were harvested by centrifugation at 5000*g* for 45 min to remove the cell debris, filtered through a 0.22-μm membrane, and dialyzed against a 10× volume of phosphate-buffered saline [10 mM phosphate (pH 7.5) and 300 mM NaCl] overnight at 4°C. The dialyzed media were supplemented with 20 mM imidazole and loaded onto a HisTrap HP (GE Healthcare) column overnight at room temperature before elution with a linear imidazole gradient (20 to 500 mM imidazole) in 25 mM tris (pH 7.5) and 500 mM NaCl. The eluted proteins were pooled together and deglycosylated with Endo_F1 overnight at 4°C, concentrated, and applied to a size-exclusion chromatography column (Superdex 200 16/600). The eluted peak corresponding to a monomeric species was concentrated to about 30 mg/ml in 10 mM Hepes (pH 7.5) and 150 mM NaCl. The proteins were flash-frozen in liquid nitrogen and stored at −80°C until further use.

Crystallization screening was carried out using sitting-drop vapor-diffusion methods in CrystalQuick 96-well plates by mixing 100 nl of protein solution with 100 nl of reservoir and equilibrated against 95 μl of reservoir. Initial screening for crystals of the intact *Tg*PLP1 MACPF domain at 30 mg/ml resulted in thin plate-like crystals in 100 mM sodium citrate tribasic dehydrate (pH 5.5) and 22% PEG-1000 (polyethylene glycol, molecular weight 1000) and diffracted to 3.4 Å resolution despite several rounds of attempted optimization. For trypsin in situ limited proteolysis, a mixture of *Tg*PLP1 MACPF domain (8 mg, 40 mg/ml) and trypsin (5 μg, 1 mg/ml; Prote-ACE, Hampton) was incubated on ice and rescreened against the Hampton crystallization conditions. The crystals appeared in 0.1 M Hepes (pH 7.5), 10% isopropanol, and 20% PEG-400 conditions and grew to full size (50 μm × 50 μm × 75 μm) in 3 days. These hexagonal crystals were used for experimental phasing using K_2_PtCl_4_. A second crystal form of the trypsinized sample had space group *C*2 in 0.1 M Hepes (pH 7.5), 10% alcohol mixture, 15% PEG-1000, 15% PEG-3350, and 15% MPD. See Table 1 for data collection and crystallographic statistics.

**Table 1:** Data collection and refinement statistics for *Tg*PLP1 MACPF domain.

	**Native_1 (trypsin)**	**Pt derivative (trypsin)**	**Native_2 (trypsin)**	**Native_3 (intact)**
**Data collection**				
Space group	*P*6_5_	*P*6_5_	*C*2	*C*222_1_
Cell dimensions				
*a*, *b*, *c* (Å)	104.86, 104.86, 52.01	105.53, 105.53, 52.72	159.49, 185.74, 104.19	95.23, 206.58, 82.74
α, β, γ (°)	90, 90, 90	90, 90, 90	90, 121.48, 90	90, 90, 90
Resolution (Å)	90.81–2.03 (2.08–2.03)	52.76–2.34 (2.40–2.34)	56.35–5.11 (5.29–5.11)	64.58–3.11 (3.16–3.11)
*R*_merge_	0.171 (1.994)	0.115 (1.901)	0.131 (1.044)	0.278 (2.132)
*R*_pim_	0.041 (0.464)	0.021 (0.341)	0.056 (0.473)	0.049 (0.596)
*I*/σ*I*	13.4 (1.6)	24.0 (2.1)	8.2 (1.5)	12.9 (1.1)
Completeness (%)	100 (20.1)	99.3 (93.2)	99.7 (100)	100 (100)
Redundancy	13.4 (1.6)	39.6 (31.3)	6.7 (6.7)	31.9 (13.4)
CC half	0.999 (0.514)	1.000 (0.444)	0.995 (0.702)	0.997 (0.582)
**Refinement**				
Resolution (Å)	98.37–2.03 (2.08–2.03)		56.35–5.11 (5.29–5.11)	64.58–3.11 (3.16–3.11)
No. of reflections	418,830 (42,406)		70,739 (7,144)	482,085 (20,145)
Unique reflections	21,264 (2,104)		10,514 (1,060)	15,081 (1,489)
*R*_work_/*R*_free_	19.11/23.25		28.60/29.73	23.26/26.46
No. of atoms				
Protein	2,144		12,864	4,552
Ligand/ion	14		84	22
*B*-factors				
Protein	55.34		330.77	101.61
Ligand/ion	51.27		363.24	143.68
RMSDs				
Bond lengths (Å)	0.002		0.003	0.003
Bond angles (°)	0.58		0.65	0.72

The glycosylated and nonglycosylated forms of *Tg*PLP1 APCβ domain were eluted at different concentrations of imidazole. The nonglycosylated form was concentrated to 21 mg/ml and crystallized in 25% PEG-3350, 0.2 M MgCl_2_, and 0.1 M bis-tris (pH 5.5). See Table 2 for data collection and crystallographic statistics.

**Table 2:** Data collection and refinement statistics for *Tg*PLP1 APCβ domain.

	**Native**	**Pt derivative**
**Data collection**		
Space group	*C*2	*P*1
Cell dimensions		
*a*, *b*, *c* (Å)	100.76, 50.21, 52.28	51.01, 52.88, 56.89
α, β, γ (°)	90, 90.96, 90	89.62, 63.34, 89.97
Resolution (Å)	50.27–1.11 (1.15–1.11)	52.88–1.51 (1.55–1.51)
*R*_merge_	0.065 (0.672)	0.038 (0.431)
*R*_pim_	0.031 (0.429)	0.018 (0.223)
*I*/σ*I*	11.4 (1.5)	30.5 (3.0)
Completeness (%)	96.8 (76.3)	60.4 (3.7)
Redundancy	6.1 (4.6)	8.6 (4.6)
CC half	0.999 (0.568)	0.999 (0.845)
**Refinement**		
Resolution (Å)	50.37–1.11 (1.15–1.11)	
No. of reflections	605,707 (33,178)	
Unique reflections	99,521 (7,980)	
*R*_work_/*R*_free_	14.93/16.67	
No. of atoms		
Protein	2,039	
Ligand/ion	437	
*B*-factors		
Protein	16.27	
Ligand/ion	33.93	
RMSDs		
Bond lengths (Å)	0.006	
Bond angles (°)	1.08	

### Crystal diffraction data collection, processing, and structure determination

All the crystals were cryocooled in liquid nitrogen with 25% glycerol as the cryoprotectant. The crystal diffraction data were collected on I04 and I24 beamlines at Diamond Light Source. To experimentally phase the MACPF domain and APCβ domain, crystals were both soaked in concentrated K_2_PtCl_6_ for 1 hour at room temperature, and the derivative data sets were collected at a wavelength of 1.0722 Å. All the diffraction data were indexed, integrated, and scaled using the Xia2 pipeline (*28*).

Experimental phasing using Pt-derivative data sets was carried out using HKL2Map (*29*). For the MACPF domain, crystals soaked with Pt diffracted to 2.3 Å and showed a strong anomalous signal, which was used to phase the structure using single-wavelength anomalous dispersion. Electron density for the Pt-derivative data set had a correlation of local RMS density of 0.84 and allowed side chains to be assigned without ambiguity. Phase extension to 2.0 Å from the native data set allowed phenix.autobuild (*30*) to trace the structure, giving a model 80% complete. The model was completed manually in Coot (*31*) and then refined in phenix.refine (*32*). The refined model was then used for the molecular replacement solution of other crystal forms in phenix.phaser (*33*). Additional density was immediately identified for the intact MACPF domain crystals despite their resolution, and to facilitate model building, multiple–data set averaging with noncrystallographic symmetry was used, resulting in clarified electron density to which missing residues could be assigned. The intact MACPF crystal data set was achieved by combining 10 different sweeps from 10 crystals. See Table 1 for MACPF domain structure determination and refinement statistics.

For the APCβ domain, the experimental phasing by platinum derivative single anomalous dispersion at 1.5 Å in phenix.autosol (*34*) resulted in an excellent electron density map with unambiguous density for side chains. This map was used in phenix.autobuild (*30*) to automatically generate a partial model. The partial model was then taken to perform molecular replacement to solve the native data set structure. The subsequent refinement was performed in Coot (*31*) and phenix.refine (*32*). See Table 2 for APCβ domain data collection and structure refinement statistics.

### Detergent-induced oligomerization

*Tg*PLP1 MACPF domain and MACPF-APCβ protein were diluted in 20 mM Hepes (pH 7.5), 150 mM NaCl, and 0.1% deoxycholate to a final concentration of 0.1 mg/ml and incubated at 37°C overnight. For SDS-PAGE analysis, 10 μl of protein samples was mixed with 5 μl of SDS-PAGE loading dye and loaded to 3 to 8% SDS-PAGE gradient gel. For analytical ultracentrifugation, the proteins were diluted in the same buffer to a final concentration of 0.5 mg/ml.

### Analytical ultracentrifugation

Sedimentation velocity experiments were performed using a Beckman Optima XL-I analytical ultracentrifuge equipped with both absorbance and interference optics. Double-sector 12-mm path-length centerpieces were used with protein samples at 0.5 mg/ml and 3-mm path length with samples at 1 mg/ml, with absorbance measurements made at 280 nm. Experiments were performed at 20°C, taking sample distribution scans every 6 min. Data were analyzed using SEDFIT software using the c(s,f/f_0_) method of interpretation to generate sample distributions in s (sedimentation coefficient) without assuming a particular number of species. The resulting distributions were curve-fit in ProFit (QuantumSoft).

### Atomistic MD simulations

All MD simulations were performed using Gromacs v5.1 (*35*, *36*). An SPC water model was used. Bond lengths and angles were constrained using the LINCS algorithm (*37*). A time step of 0.002 ps was used, writing atomic coordinates every 10 ps. Temperature was maintained at 310 K using velocity rescaling, with a coupling constant of 1 ps (*38*). In simulations containing lipids, the solvent, protein, and lipid were separately coupled to an external water bath. Isotropic pressure coupling was used to set the pressure to 1 bar using the Parrinello-Rahman barostat (*39*), with a coupling constant of 1 ps. Coulombic forces were treated using Particle-Mesh Ewald electrostatics (*40*). Van der Waals and electrostatics were shifted to zero at the cutoff of 1 nm.

Proteins were solvated and NaCl ions were added to neutralize the system and create a salt concentration of 0.1 M. Systems were subjected to up to 5000 steps of energy minimization using the steepest descent algorithm. The system was equilibrated by restraining positions of protein heavy atoms using a force constant of 1000 kJ mol^−1^ nm^−3^ for 1 ns. The position restraints were then removed, and final production runs of 100 ns were performed. Three repeats were produced for each simulation, each starting with random velocities.

MODELLER (*41*) was used to connect free termini of *Tg*PLP1 MACPF domain. Simulations were prepared using the Amber 99SB-ILDN force field (*42*).

Simulations of *Tg*PLP1 APC-ß domain membrane binding were performed using the Gromos 53a6 force field (*43*). For atomistic simulations of *Tg*PLP1 APCβ bound to a membrane bilayer, coarse-grained simulations provided an initial configuration of APCβ membrane binding, which was then converted to an atomistic structure for further simulations and analysis in greater detail following the conversion protocol set out by Stansfeld and Sansom (*44*).

### Coarse-grained MD simulations

For coarse-grained simulations, the Martini v2.2 coarse grain force field was used (*45*, *46*). Lennard-Jones interactions were shifted to zero between 0.9 and 1.1 nm. The electrostatic potential energy was shifted to zero between 0 and 1.1 nm. The system was kept at a constant temperature of 323 K coupled using velocity rescaling (*38*), with protein, water, ions, and lipids coupled separately. The pressure was coupled semi-isotropically using the Berendsen algorithm (*47*) and maintained at 1 bar with a compressibility of 5 × 10^−6^. The coupling constant values for temperature and pressure were 1 and 4 ps, respectively. The time step for integration was 20 fs, and particle coordinates were written to the trajectory file every 200 ps.

Elastic network modelling was applied using a force constant of 1000 kJ mol^−1^ nm^−2^. The lower elastic bond cutoff used was 0.5 nm, and the upper cutoff was 1.0 nm.

Simulations were produced using a protocol similar to that used by Yamamoto *et al.* (*48*). The APCβ domain was placed 10 nm away from a preformed bilayer. The system was solvated with water. NaCl ions (150 mM) were added. Energy minimization was performed for 300 steps before starting production runs of 1 μs, allowing the protein to diffuse in the box and encounter the membrane.

Lipids POPC, POPE, POPS, PIP_2_, and PIP_3_ were used to form membrane bilayers of the following compositions: 100% POPC; 100% POPE; 80% POPC and 20% POPS; 45% POPC, 45% POPE, and 10% POPS; 45% POPC, 42% POPE, 10% POPS, and 3% PIP_2_; and 45% POPC, 42% POPE, 10% POPS, and 3% PIP_3_. Ensembles of 20 μs × 1 μs simulations were set up for each bilayer type. The protein starting orientation was sequentially rotated around the *X*, *Y*, and *Z* coordinates in each simulation of the ensemble to reduce bias.

Membrane-binding orientations of *Tg*PLP1 APCβ domain were investigated by representing two-dimensional histograms of distance between protein and membrane center of mass against the YY component of the rotational matrix of APCβ in membrane association simulations. Rotation and translation in the *x* and *y* plane of the simulation were first removed, and then the rotational matrix required for least-squares fitting of APCβ onto a reference structure using gmx rotmat was calculated. As the APCβ domain was positioned at a different orientation in each simulation, the rotational matrix was calculated against a starting orientation of 0° on each of *X*, *Y*, and *Z* matrix components for direct comparison of orientation.

### Small-angle x-ray scattering

SAXS experiments were carried out in 10 mM tris (pH 7.5) and 150 mM NaCl with deglycosylated *Tg*PLP1 (residues 462 to 1072, MACPF and APCβ domain) at three different concentrations: 1.5, 5, and 8 mg/ml. SAXS data were collected in BM29 at the ESRF with a momentum transfer range of 0.004 Å^−1^ < *q* < 0.45 Å^−1^, where *q* = 4πsin(θ)/λ and 2θ is the scattering angle. The scattering intensity from buffer alone was subtracted from the averaged sample data to obtain the protein scattering in solution. The radius of gyration (*R*_g_) was calculated from a Guinier plot using AutoRg (*49*). The particle distance distribution function P(r) was calculated in GNOM (*50*) using a low-resolution data range (0.01 Å^−1^< *q* < 0.15 Å^−1^). Data reduction was carried out using the ATSAS package (*49*).

The combination of the MACPF and APCβ was modeled into the SAXS data using the online server MultiFoXS (http://modbase.compbio.ucsf.edu/multifoxs/). Briefly, the disordered loops within the MACPF domain and a polyhistidine tag at the C terminus of the APCβ domain were first modeled in MODELLER. Then, the 10 amino acid residues (residues 800 to 809), which were absent in both structures, were treated as a flexible linker between the MACPF domain and APCβ domain acting as rigid bodies. In total, about 10,000 models were generated (*51*), and the SAXS profile was then calculated for each conformation using the FoXS method (*52*). The multistate models were enumerated in MultiFoXS using the branch-and-bound method. To characterize the range of conformations consistent with the SAXS data, we analyzed distributions of *R*_g_ for the entire ensemble of 10,000 models and the 1000 best-scoring *N*-state models (*N* = 1… 5).

### Molecular graphics

Molecular structures are displayed using PyMOL (The PyMOL Molecular Graphics System, Version 2.0 Schrödinger, LLC), Chimera (*53*), and VMD (*54*).

### Liposome-binding assay

PIP_3_ was prepared in chloroform/methanol (8:2), and other lipids were dissolved in chloroform. Lipids (2 mg) with different compositions were mixed and dried in a clean Pyrex tube at room temperature. Residual chloroform was then removed in a desiccator attached on a CARIO-SP diaphragm pump overnight. The lipid film was rehydrated by addition of 1 ml of solubilization buffer [20 mM Hepes (pH 7.5) and 150 mM NaCl] followed by vigorous vortexing. The hydrated lipid film was then extruded through 100-nm pore membranes 11 times. The quality of liposomes was checked by dynamic light scattering, and they were used within 2 days. To perform liposome sedimentation assays, 100 μl of liposomes at 2 mg/ml was mixed with *Tg*PLP1 MACPF-APCβ or APCβ for 2 hours at room temperature. The liposome-protein mixture was then centrifuged at 67,000 rpm in an ultracentrifuge Optima TL with a TLA100.4 rotor for 20 min. The resulting supernatant was removed, and liposome-protein pellets were washed with 100 μl of solubilization buffer and centrifuged again for 20 min at 67,000 rpm. The pellets were then dissolved in 10 μl of solubilization buffer with 5 μl of SDS-PAGE loading buffer. Supernatant (10 μl) was loaded as well to check the unbound protein in solution. The liposome sedimentation assay was performed at least three times.

### Negative-stain electron microscopy

*Tg*PLP1 MACPF-APCβ domain tandem or MACPF domain alone preincubated with deoxycholate was placed on a C-flat carbon-coated electron microscopy grid and stained using uranyl formate. The samples were imaged using an FEI T12 electron microscope operating at 120 kV.

### GUV electroformation and imaging

The electroformation method was used for GUV preparation, basically as described in the study of Ruan *et al.* (*25*). The lipid mixture used [POPS (1-palmitoyl-2-oleoyl-*sn*-glycero-3-phospho-l-serine)/POPE (1-palmitoyl-2-oleoyl-*sn*-glycero-3-phosphoethanolamine)/POPC (1-palmitoyl-2-oleoyl-*sn*-glycero-3-phosphocholine)/rhodamine-DHPE (Lissamine rhodamine B 1,2-dihexadecanoyl-*sn*-glycero-3-phosphoethanolamine, triethylammonium salt), 10:40:49.5:0.5 (mol/mol)] and was dissolved in chloroform. The lipid mixture was spread on the conductive site of an ITO slide, and dried under vacuum. Electroformation was carried out in sucrose solution [300 mM sucrose and 1 mM Hepes (pH 7.4)] between two conductive ITO slides (Vesicle Prep Pro, Nanion Technologies). The following electroformation protocol with ac current was applied: initial increase of amplitude from 0 to 3 V with 5-Hz frequency, followed by 2 hours of constant amplitude 3 V and frequency 5 Hz, and ending with decreasing frequency from 5 to 0 Hz by constant amplitude 3 V. After electroformation, GUVs were sedimented by addition of glucose solution [300 mM glucose, 1 mM Hepes (pH 7.4)] and outside buffer [10 mM Hepes, 150 mM NaCl (pH 7.4)]. GUVs were left to sediment overnight. The osmolaritiy of all solutions used for GUV preparation and experiments was adjusted by using an osmometer Osmomat 3000 (Gonotec GmbH). GUVs were used immediately after sedimentation. To perform GUV-binding assays, GUVs were mixed with the *Tg*PLP1 C-terminal APCβ domain (100 μg/ml final concentration). After incubation for 30 min at room temperature in the dark, 0.5 μg of anti-His_6_ antibodies labeled with Alexa Fluor 488 (Thermo Fisher Scientific) was added to the GUVs and proteins. After an additional 30-min incubation at room temperature in the dark, images were recorded on a Leica TCS SP5 laser scanning microscope with a 63× oil immersion objective (numerical aperture, 1.4). The rhodamine-containing GUV membrane was excited at 543 nm, and emission was detected from 570 to 620 nm. Alexa Fluor 488–labeled antibodies were excited at 488 nm, and emission was detected from 510 to 530 nm. As a control, GUVs and antibodies without protein were mixed and incubated for 60 min at room temperature in the dark before imaging.

### Planar lipid bilayer formation and recording of conductances

For all experiments, planar lipid bilayers were formed from a mixture of POPS/POPE/POPC = 1:4:5 (mol/mol/mol) dissolved in octane with a final concentration of lipids (5 mg/ml). For electrical measurements in planar lipid bilayers, an integrated chip-based recording setup Orbit mini and EDR2 software (Nanion Technologies) were used. Recordings were obtained in parallel with multielectrode-cavity-array chips (Ionera Technologies). The aqueous phase was composed of 20 mM Hepes and 500 mM NaCl (pH 7.4). Proteins were added to the cis side of the bilayer to a final concentration of ~0.1 mg/ml. To promote pore insertion, a voltage of +150 mV with a 20-kHz sampling rate was applied. All measurements were done at room temperature. Clampfit (10.7.0.3.) was used to analyze current traces.

## Supplementary Material

http://advances.sciencemag.org/cgi/content/full/4/3/eaaq0762/DC1

## SUPPLEMENTARY MATERIALS

Supplementary material for this article is available at http://advances.sciencemag.org/cgi/content/full/4/3/eaaq0762/DC1 fig. S1. Domain architecture of ApiPLPs. fig. S2. Structure of *Tg*PLP1 MACPF domain in its helical assembly. fig. S3. Analysis of the predicted conductance properties of *Tg*PLP1 oligomers using the program HOLE. fig. S4. Analysis of the structural stability of *Tg*PLP1 MACPF crystal structures during MD simulation. fig. S5. Comparison of *Tg*PLP1 MACPF oligomeric crystal structures with atomistic simulations showing changes in intersubunit hydrogen bonding. fig. S6. Normalized bar charts showing main intersubunit hydrogen bonds during *Tg*PLP1 MACPF helix and ring simulations. fig. S7. Simulation and hydrogen bond analysis of representative ring and helix interfaces isolated from oligomers to remove oligomeric constraints. fig. S8. Details of *Tg*PLP1 APCβ domain crystal structure. fig. S9. Details of coarse-grained and atomistic *Tg*PLP1 APCβ-membrane simulations. fig. S10. AUC and SAXS study of *Tg*PLP1 (MACPF-APCβ).
