# Supplementary material for: Structures of monomeric and oligomeric forms of the Toxoplasma gondii perforin-like protein 1
Source: Sci Adv. 2018 Mar 21;4(3):eaaq0762. doi: 10.1126/sciadv.aaq0762 (PMC5943054; doi:10.1126/sciadv.aaq0762)
Supplement: http://advances.sciencemag.org/cgi/content/full/4/3/eaaq0762/DC1 [file supp_4_3_eaaq0762__index.html]

Science Advances | Science Advances

## Supplementary Materials

**This PDF file includes:**

- fig. S1. Domain architecture of ApiPLPs.
- fig. S2. Structure of *Tg*PLP1 MACPF domain in its helical assembly.
- fig. S3. Analysis of the predicted conductance properties of *Tg*PLP1 oligomers using the program HOLE.
- fig. S4. Analysis of the structural stability of *Tg*PLP1 MACPF crystal structures during MD simulation.
- fig. S5. Comparison of *Tg*PLP1 MACPF oligomeric crystal structures with atomistic simulations showing changes in intersubunit hydrogen bonding.
- fig. S6. Normalized bar charts showing main intersubunit hydrogen bonds during *Tg*PLP1 MACPF helix and ring simulations.
- fig. S7. Simulation and hydrogen bond analysis of representative ring and helix interfaces isolated from oligomers to remove oligomeric constraints.
- fig. S8. Details of *Tg*PLP1 APCβ domain crystal structure.
- fig. S9. Details of coarse-grained and atomistic *Tg*PLP1 APCβ-membrane simulations.
- fig. S10. AUC and SAXS study of *Tg*PLP1 (MACPF-APCβ).

Download PDF

**Files in this Data Supplement:**

- Adobe PDF - aaq0762\_SM.pdf
