# Supplementary material for: Structures of monomeric and oligomeric forms of the Toxoplasma gondii perforin-like protein 1
Source: Sci Adv. 2018 Mar 21;4(3):eaaq0762. doi: 10.1126/sciadv.aaq0762 (PMC5943054; doi:10.1126/sciadv.aaq0762)
Supplement: http://advances.sciencemag.org/cgi/content/full/4/3/eaaq0762/DC1 [file aaq0762_SM.pdf]

Science Advances

AAAS

advances.sciencemag.org/cgi/content/full/4/3/eaaq0762/DC1

Supplementary Materials for

Structures of monomeric and oligomeric forms of the *Toxoplasma gondii* perforin-like protein 1

Tao Ni, Sophie I. Williams, Saša Rezelj, Gregor Anderluh, Karl Harlos, Phillip J. Stansfeld, Robert J. C. Gilbert

Published 21 March 2018, *Sci. Adv.* **4**, eaaq0762 (2018)
DOI: 10.1126/sciadv.aaq0762

This PDF file includes:

- fig. S1. Domain architecture of ApiPLPs.
- fig. S2. Structure of *Tg*PLP1 MACPF domain in its helical assembly.
- fig. S3. Analysis of the predicted conductance properties of *Tg*PLP1 oligomers using the program HOLE.
- fig. S4. Analysis of the structural stability of *Tg*PLP1 MACPF crystal structures during MD simulation.
- fig. S5. Comparison of *Tg*PLP1 MACPF oligomeric crystal structures with atomistic simulations showing changes in intersubunit hydrogen bonding.
- fig. S6. Normalized bar charts showing main intersubunit hydrogen bonds during *Tg*PLP1 MACPF helix and ring simulations.
- fig. S7. Simulation and hydrogen bond analysis of representative ring and helix interfaces isolated from oligomers to remove oligomeric constraints.
- fig. S8. Details of *Tg*PLP1 APCβ domain crystal structure.
- fig. S9. Details of coarse-grained and atomistic *Tg*PLP1 APCβ-membrane simulations.
- fig. S10. AUC and SAXS study of *Tg*PLP1 (MACPF-APCβ).

Supplementary Figures

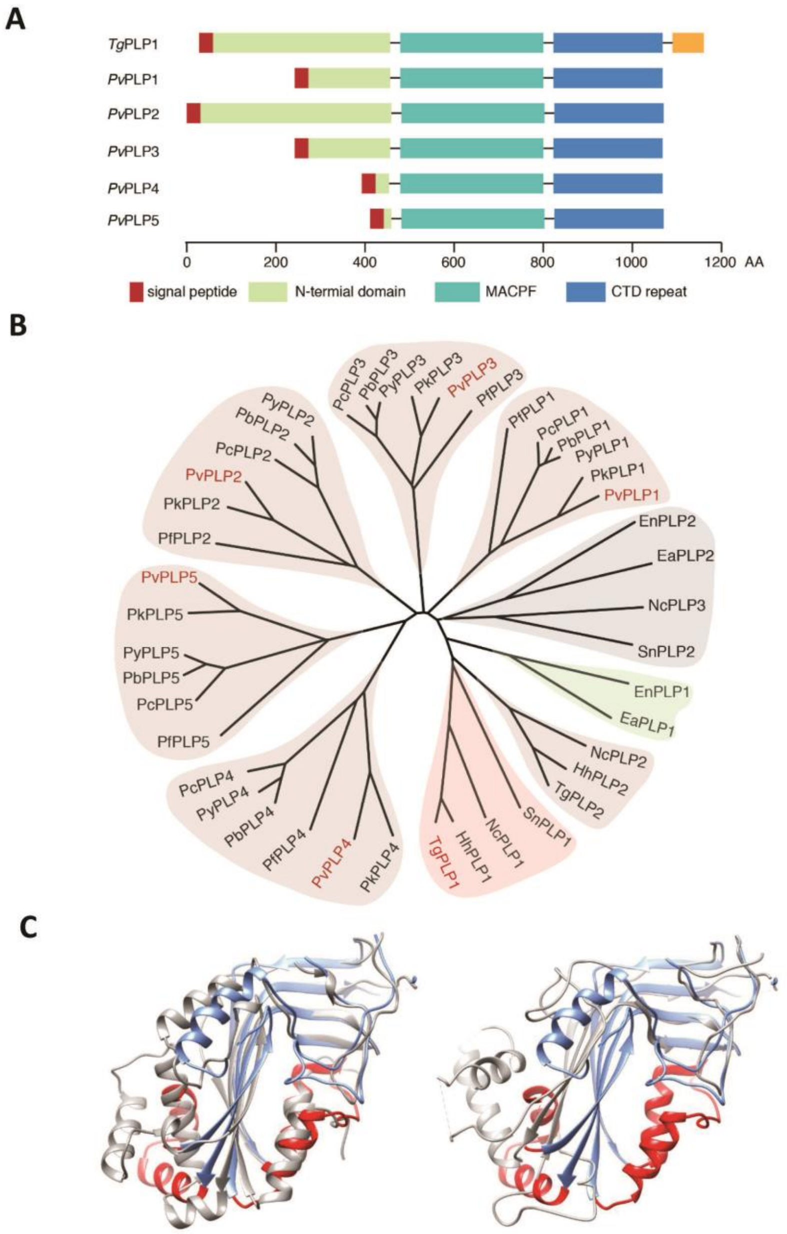

fig. S1. Domain architecture of ApiPLPs. (**A**)Schematic domain architecture of PLPs from *Toxoplasma gondii* and *Plasmodium vivax*. PLPs share conserved MACPF domains and C-terminal APCβ domains, with wide variance in their N-terminal domains. *Tg*PLP1 has an extra ~80 amino acids at the very C-terminal end, depicted in yellow. (**B**) Phylogeny of ApiPLPs based on protein sequence. For simplicity, only PLPs from *Plasmodium spp.* and representative *Toxoplasma spp.* were used in construction of the phylogenetic tree. Protein sequence alignment was performed in Clustal Omega. Abbreviations: Pf: *Plasmodium falciparum*, Pk: *Plasmodium knowlesi*, Pv: *Plasmodium vivax, Pb: Plasmodium berghei*, *Pc: Plasmodium chabaudi*, Py: *Plasmodium yoelli.* (**C**) The two transmembrane hairpin (TMH) regions of PLPs are largely helical in their precursor structures. Pore-forming proteins such as lymphocyte perforin-1 have TMHs of ~50 residues; *Tg*PLP1 (this paper) ~45; the TMHs of non-pore-forming astrotactin-2 are mismatched at 19 and 54 residues, respectively (*11*, *15*). On the left here are shown superpositions of relevant regions from perforin-1 (grey) and *Tg*PLP1 (this work – TMHs colored red). On the right a similar superposition for astrotactin-2 and *Tg*PLP1.

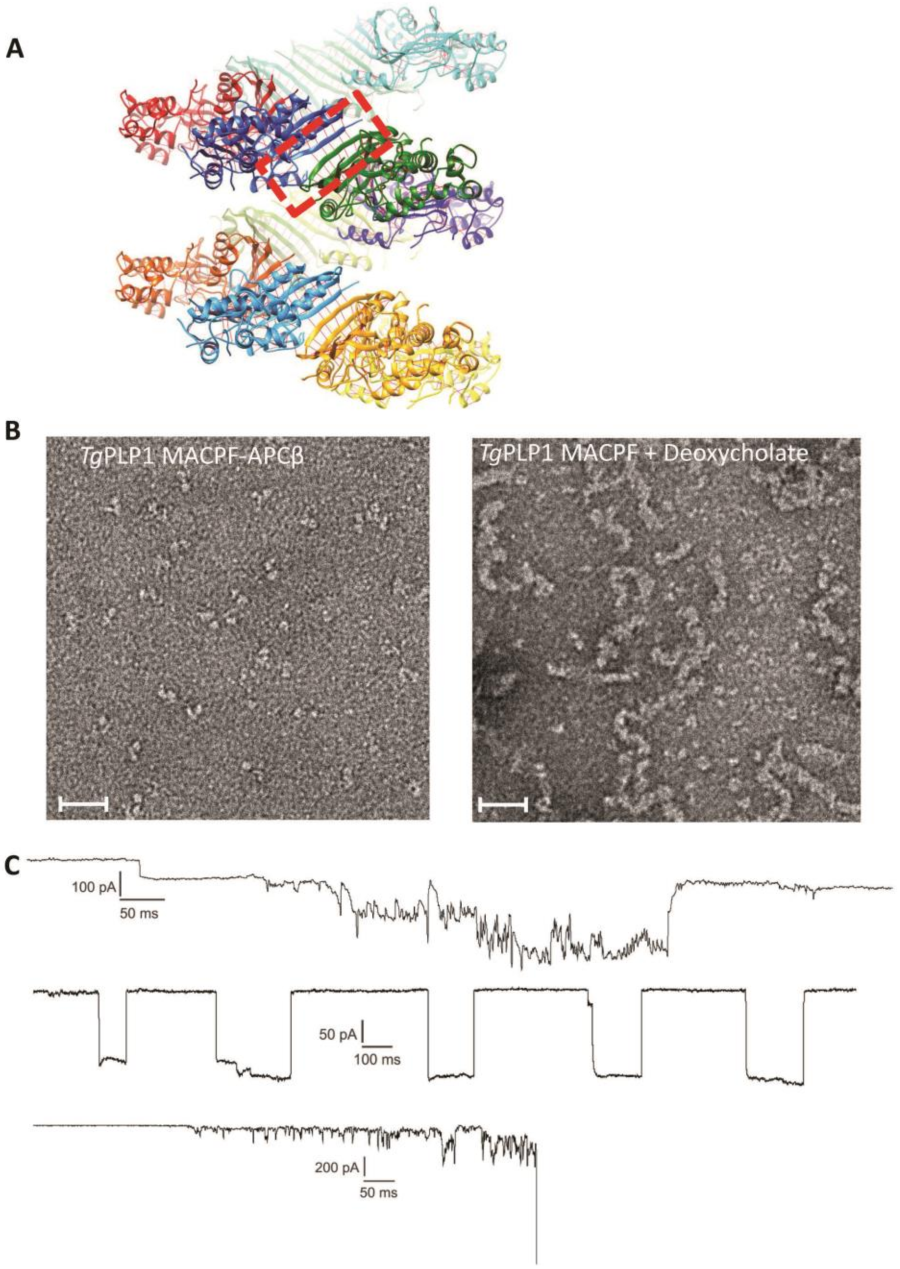

fig. S2. Structure of *Tg*PLP1 MACPF domain in its helical assembly. (**A**) The structure is colored according to the individual domains. The hydrogen bonds between and within MACPF domains are shown using red lines. The hydrogen bonds between one domain interface are highlighted with a red box. (**B**) Negative stain imaging of monomeric *Tg*PLP1 MACPF-APCβ tandem construct (left) and of oligomers formed by the MACPF domain in the presence of deoxycholate (right). Scale bar = 20 nm. (**C**) Conductances induced by full-length *Tg*PLP1 oligomers in a planar lipid bilayer system. The oligomers were formed by treatment with deoxycholate followed by dialysis. The top trace shows several conductance events of variable size and stability; the middle trace shows a single well-defined pore undergoing sporadic closure events; the bottom trace shows another frequently-observed phenomenon – membrane breakage.

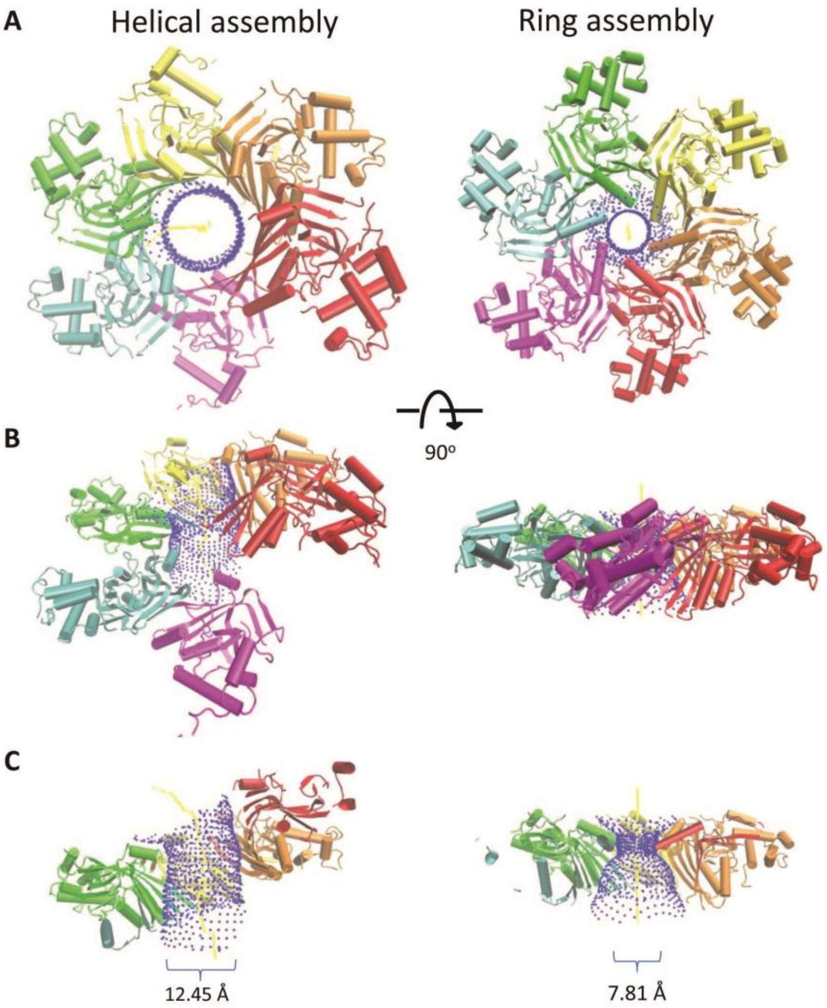

fig. S3. Analysis of the predicted conductance properties of *Tg*PLP1 oligomers using the program HOLE. Top view (**A**), side view (**B**) and cross-section of side view (**C**) of the helical assembly and ring assembly, respectively. The blue dots indicate the solvent boundary within a pore generated by a helix or ring of subunits. The yellow line indicates the center of the inferred pore. As calculated using the program HOLE *(19)* for the helical assembly the minimal functional radius, R^e_{min} is 12.45 Å and the inferred macroscopic conductance in 1M KCl (G_{macro}) is 12.0 nS; for the ring R^e_{min} = 7.80 Å and G_{macro} = 8.9 nS.

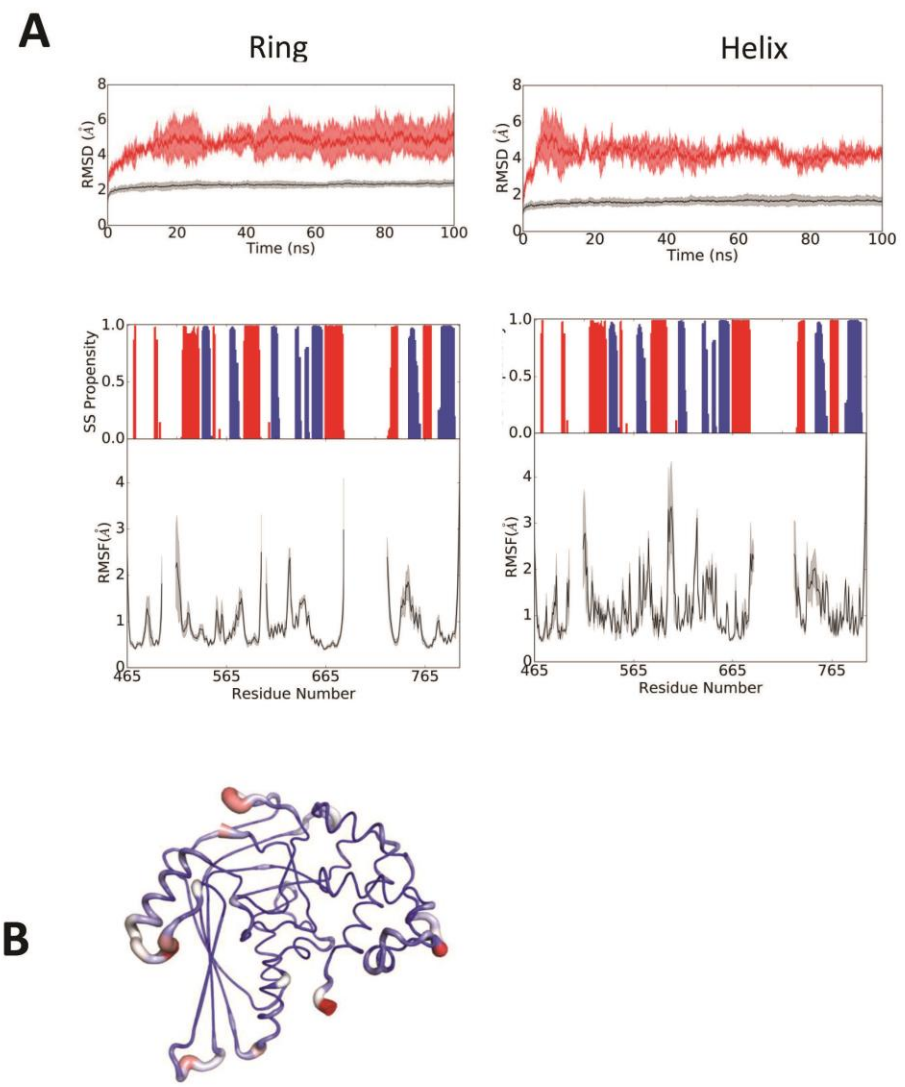

fig. S4. Analysis of the structural stability of *Tg*PLP1 MACPF crystal structures during MD simulation. (**A**) RMSD and RMSF plots of the *Tg*PLP1 MACPF hexameric ring and helix showing structural stability from 3 replicates of 100 ns atomistic simulations. RMSDs were calculated for each MACPF subunit (black line), and for the whole molecule (red line). The mean value of each repeat is plotted along with standard deviation to indicate overall structural flexibility. RMSF was calculated for each MACPF subunit and the mean and standard deviation are plotted to show structural flexibility on a residue by residue basis. Secondary structure propensity across the structure was calculated throughout the simulations, and represented in blue as α-helix and red as β-strand. (**B**) Cartoon representation of *Tg*PLP1 MACPF subunit indicating regions of structural stability during simulations. Degree of flexibility is represented from a scale of red to blue, and by thickness of line, with red color and thicker lines indicating high flexibility.

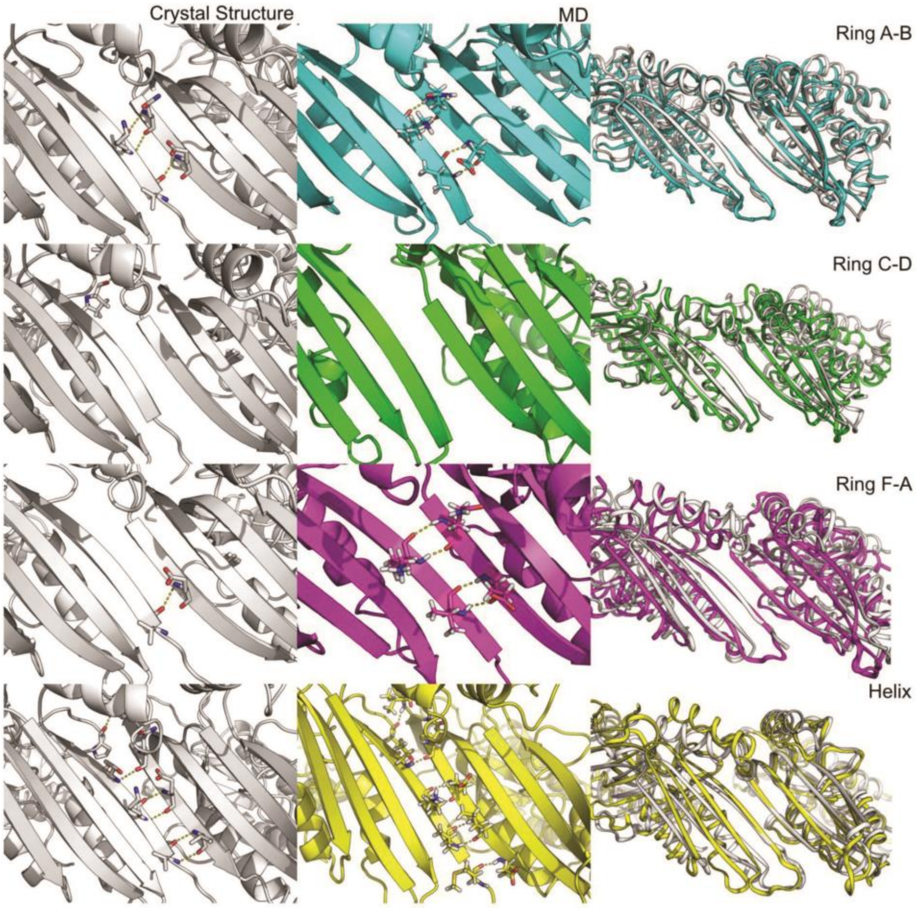

fig. S5. Comparison of *Tg*PLP1 MACPF oligomeric crystal structures with atomistic simulations showing changes in intersubunit hydrogen bonding. Cartoon representations of representative subunit interfaces from *Tg*PLP1 MACPF oligomeric ring and helix crystal structures and snapshots from atomistic simulations of the structures are shown alone, and aligned. In each case the crystal structure is colored grey. Snapshots are representative of main chain hydrogen bonds identified in hydrogen bond analysis from 3 replicates of each simulation (see fig. S6).

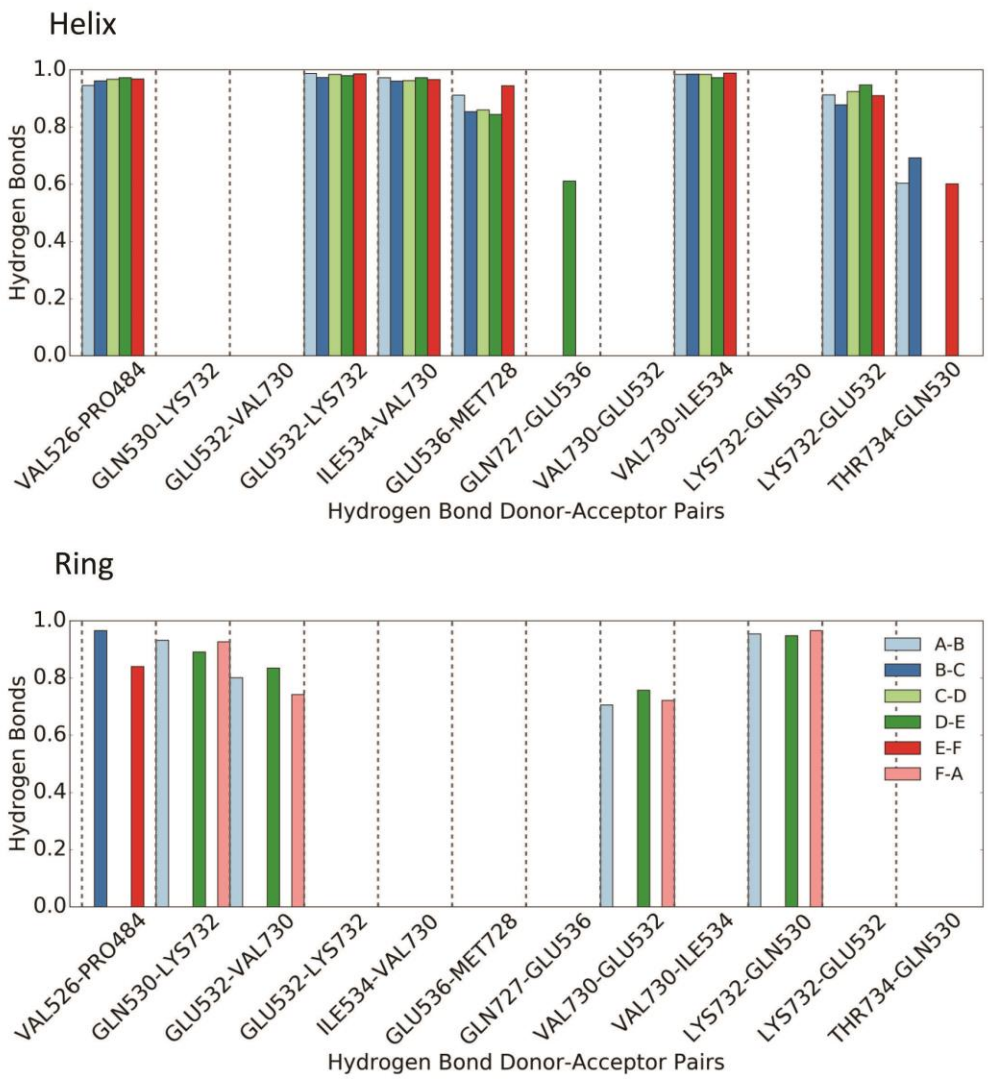

fig. S6. Normalized bar charts showing main intersubunit hydrogen bonds during *Tg*PLP1 MACPF helix and ring simulations. *Tg*PLP1 MACPF ring and helix structures were simulated for 100 ns with three replicates. Hydrogen bonds present between each subunit were identified, and frequency was calculated across the simulations (number of simulation frames present). Side chain interactions were excluded and only backbone hydrogen bonds present in over 50 % of the total simulation time were plotted. The first residue listed per hydrogen bonding pair is the hydrogen bond donor. Interfaces in the helix were highly similar to each other, and well conserved throughout the simulation, while ring interfaces varied significantly.

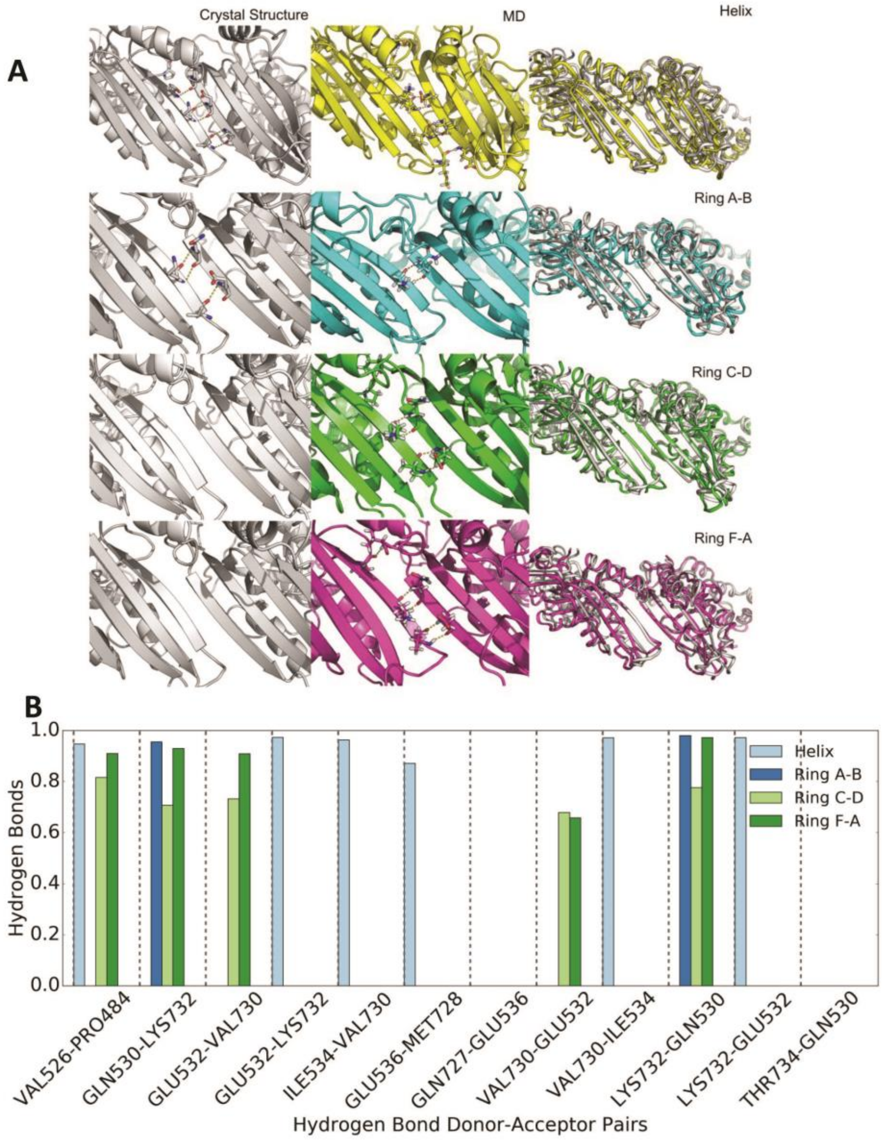

fig. S7. Simulation and hydrogen bond analysis of representative ring and helix interfaces isolated from oligomers to remove oligomeric constraints. To indicate the stability of each MACPF ring and helix interface, dimers representing each class of *Tg*PLP1 MACPF interface were modelled to represent that interface without constraint of the hexameric structures, and simulated for 100 ns. Comparisons of MACPF interfaces in crystal structures and after 100 ns atomistic simulations. Simulation snapshots of each interface are shown next the equivalent crystal structure, and aligned with the crystal structure indicating changes in inter-subunit hydrogen bonding. The crystal structure is shown in grey in each case. Normalised bar chart showing frequency of inter-subunit hydrogen bonds in each dimer, counted in simulation frames, during the course of 100 ns simulation

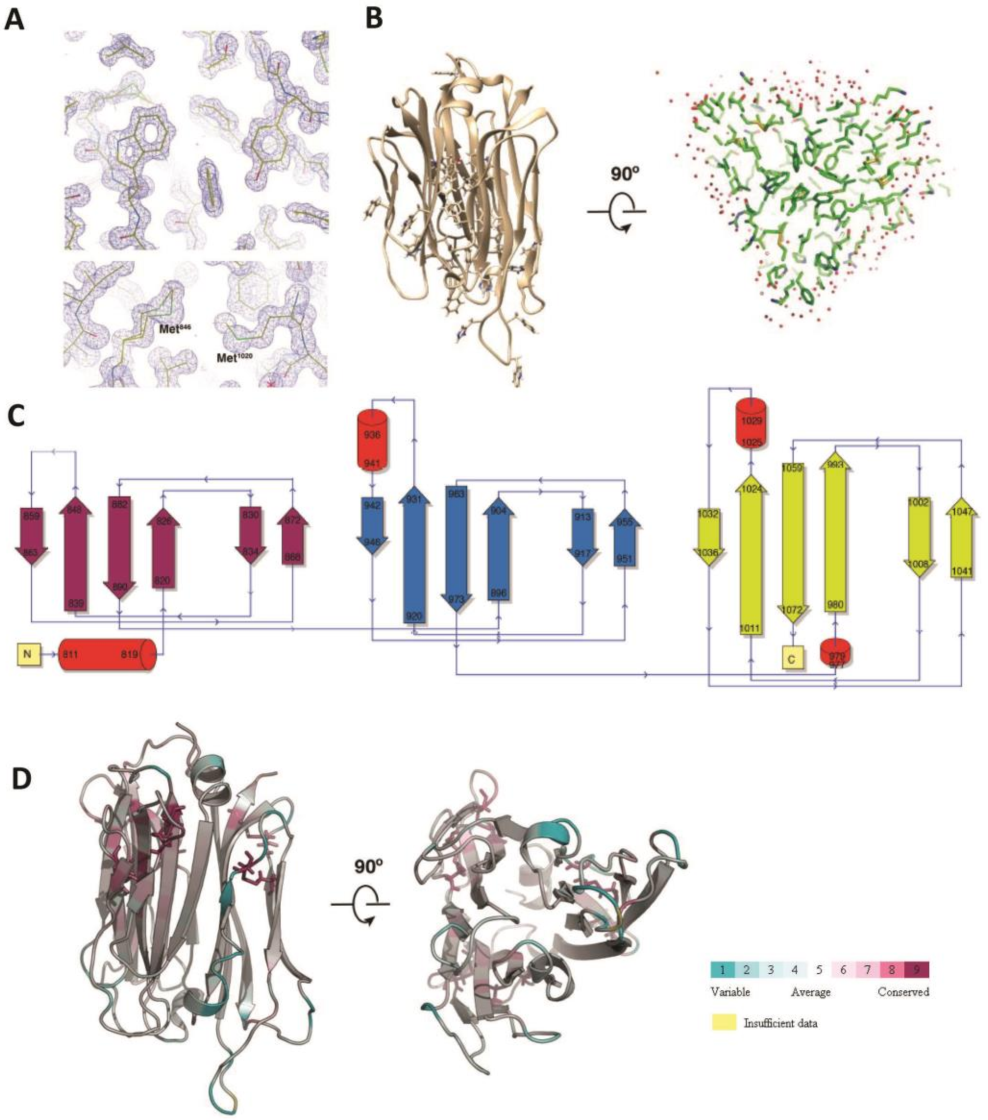

fig. S8. Details of *Tg*PLP1 APCβ domain crystal structure. (**A**) Representative electron density map of several hydrophobic residues in the core and unambiguous density of a methionine (Met^{846}) double conformation. (**B**) Left: side view of *Tg*PLP1 APCβ domain, highlighting the side chains of tryptophan, histidine and tyrosine. Right: cross-section of top view showing the hydrophobicity of the core; only the side chains of the protein and water molecules (in red dots) are shown here. (**C**) Topology diagram of TgPLP1 APCβ. There are a total of 18 β-strands divided in 3 groups, each group with 6 β-strands arranged in 2 β-sheets (4+2). The figure was prepared using the PDBSUM server (http://www.ebi.ac.uk/thornton-srv/databases/cgi-bin/pdbsum/GetPage.pl?pdbcode=index.html) with modification. (**D**) Mapping sequence conservation of apicomplexan APCβ domains to the *Tg*PLP1 APCβ domain structure. The conserved cysteines forming disulphide bondS are shown. Conservation score (0-9, colored from blue to red) was calculated with the ConSurf server (http://consurf.tau.ac.il/2016/.

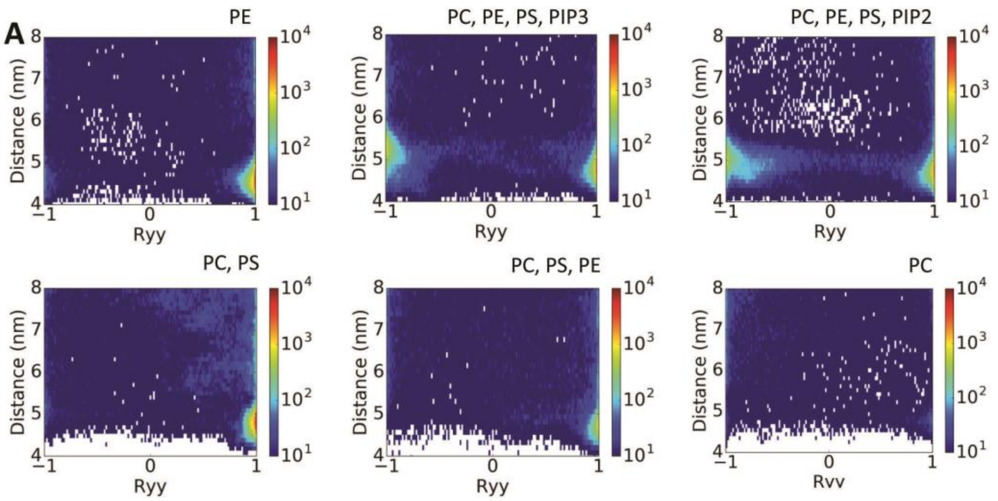

B

	Total membrane binding	Total 'upright binding'	Total 'upside down' binding
POPC	1	1	0
POPE	15	15	0
PC PS	5	5	0
PC PS PE	3	3	0
PC PE PS PIP3	16	7	9
PC PE PS PIP2	20	9	11

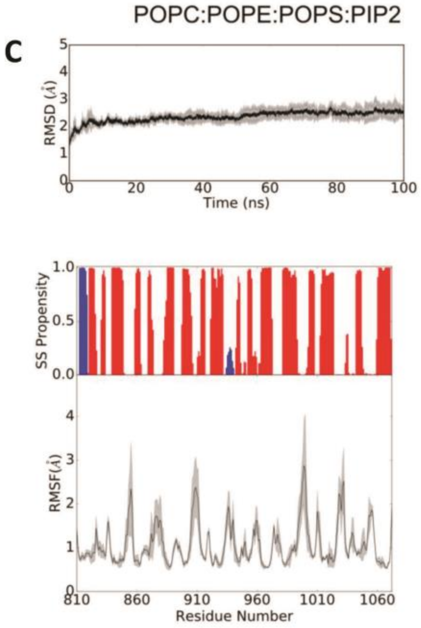

fig. S9. Details of coarse-grained and atomistic *Tg*PLP1 APCβ-membrane simulations. (**A**) Normalised density maps (log scale) for coarse grained *Tg*PLP1 APCβ membrane binding simulations indicating binding capacity and orientation within different lipid bilayer compositions. The APCβ domain was placed 7 nm away from each bilayer in a simulation box, and simulated for 1 μs allowing the protein to drift and subsequently interact with the membrane. An ensemble of 20 repeat simulations was performed for each bilayer composition. The YY component of the rotational matrix is shown against the distance between the centre of masses of the APCβ domain and lipid bilayer. (**B**) Table indicating the total number of simulations out of 20 in which *Tg*PLP1 APCβ bound to the membrane for each bilayer composition, followed by the number of simulations in which each binding orientation was observed, as defined by position at the end of the 1 μs simulation. (**C**) RMSD, RMSF and secondary structure propensity of *Tg*PLP1 APCβ bound to 45 % POPC, 42 % POPE, 10 % POPS, 3 % PIP2 bilayer in 100 ns atomistic simulation. Mean values and standard deviation for RMSD and RMSF were calculated from 3 repeat atomistic simulations.

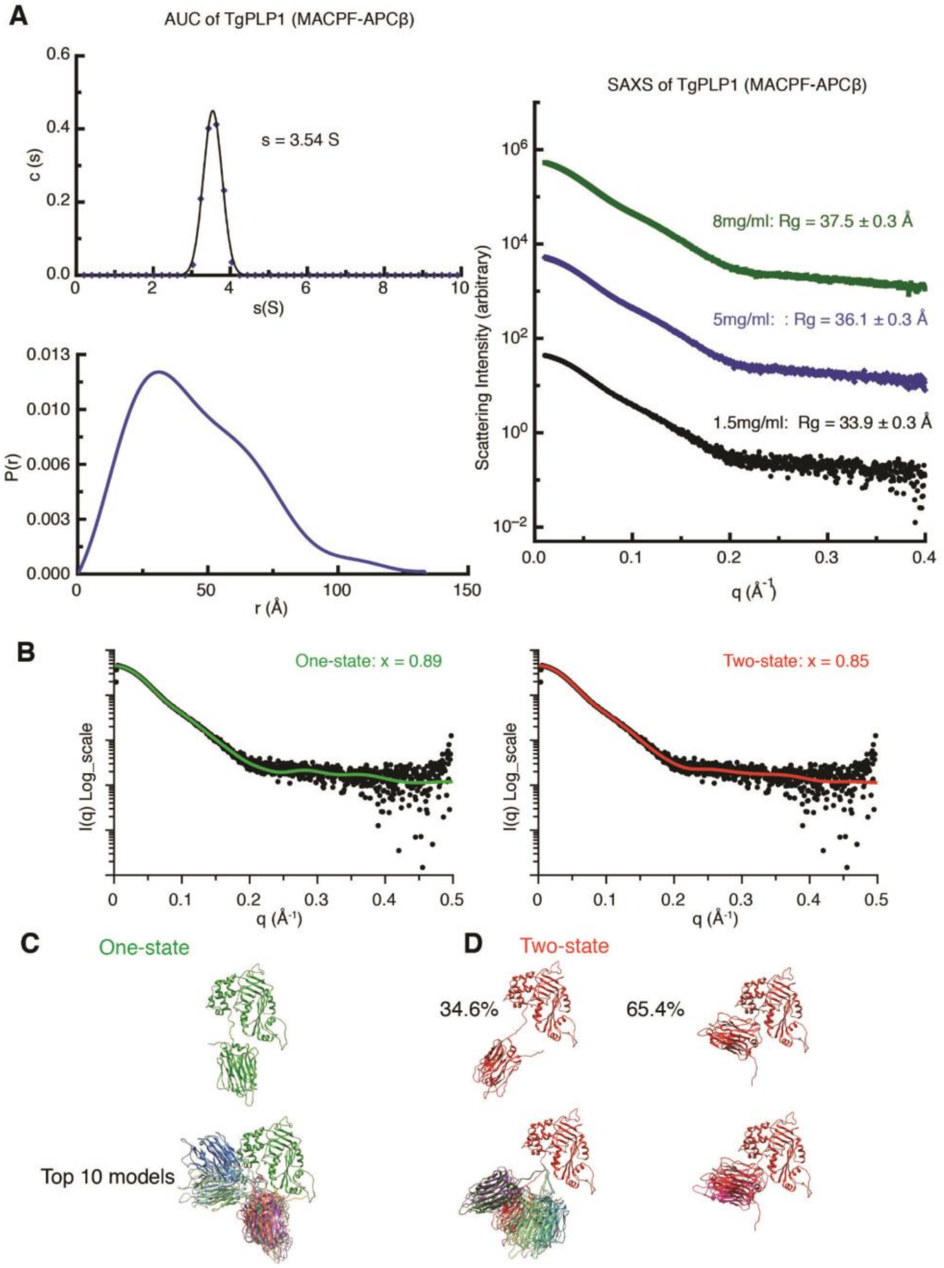

fig. S10. AUC and SAXS study of *Tg*PLP1 (MACPF-APCβ). (**A**) Top left, AUC analysis of *Tg*PLP1 MACPF-APCβ at 1 mg/ml showing the monomeric property of the protein in solution, with sedimentation coefficient of 3.54 S. Right, SAXS data for *Tg*PLP1 MACPF-APCβ at three different concentrations: 1.5, 5 and 8 mg/ml. Bottom left: distance distribution function (P(r)) of *Tg*PLP1 MACPF-APCβ at 5 mg/ml. (**B**) Comparison of the experimental SAXS profiles (black dots) with computed SAXS profiles for the top-scoring single-state (left) and two-state (right) models. (**C**) Conformations of the top-scoring single-state models are shown. The top 10 models are aligned on their MACPF domains. (**D**) Conformations of the top-scoring two-state models are shown. The top 10 models are aligned on their MACPF domains.
